# Effects of dietary fatty acids and cholesterol excess on liver injury: A lipidomic approach

**DOI:** 10.1016/j.redox.2016.09.002

**Published:** 2016-09-09

**Authors:** Gaetano Serviddio, Francesco Bellanti, Rosanna Villani, Rosanna Tamborra, Chiara Zerbinati, Maria Blonda, Marco Ciacciarelli, Giuseppe Poli, Gianluigi Vendemiale, Luigi Iuliano

**Affiliations:** a CURE University Centre for Liver Disease Research and Treatment, Institute of Internal Medicine, Department of Medical and Surgical Sciences, University of Foggia, 71122 Foggia, Italy; b Laboratory of Vascular Biology and Mass Spectrometry, Department of Medico-Surgical Sciences and Biotechnologies, Sapienza University of Rome, 04100 Latina, Italy; c Department of Clinical and Biological Sciences, University of Torino at San Luigi Gonzaga Hospital, 10043 Torino, Orbassano, Italy

**Keywords:** NAFLD, Non-Alcoholic Fatty Liver Disease, NAFL, Non-Alcoholic Fatty Liver, NASH, Non-Alcoholic SteatoHepatitis, FA, fatty acids, TG, triglycerides, SFA, saturated fatty acids, CYP, cytochrome, HFA, high fatty acids, HCh, high cholesterol, ALT, alanine aminotransferase, AST, aspartate aminotransferase, FFA, free fatty acids, 7α-OHC, 7α-hydroxycholesterol, 7β-OHC, 7β-hydroxycholesterol, 27-OHC, 27-hydroxycholesterol, 25-OHC, 25-hydroxycholesterol, 4β-OHC, 4β-hydroxycholesterol, 5β, 6β-epoxy, 5β,6β-epoxycholesterol, 5α, 6α-epoxy, 5α,6α-epoxycholesterol, triol, 5α-cholestane-3β,5,6β-triol, 7-KC, 7-ketocholesterol, 6-oxo, 6-oxo-cholestan-3β,5α-diol, CT, threshold cycle, SDM, standard deviation of the mean, ANOVA, analysis of variance, MUFA, monounsaturated fatty acids, PUFA, polyunsaturated fatty acids, CYP7A1, 7α-hydroxylase, CYP27A1, 27-hydroxylase, CYP25A1, 25-hydroxylase, CYP3A1, 4β-hydroxylase, EFA, essential fatty acids, Oxysterols, Non-alcoholic fatty liver disease, Cholesterol excess, Fatty acids

## Abstract

Lipid accumulation is the hallmark of Non-alcoholic Fatty Liver Disease (NAFLD) and has been suggested to play a role in promoting fatty liver inflammation. Previous findings indicate that during oxidative stress conditions excess cholesterol autoxidizes to oxysterols. To date, the role of oxysterols and their potential interaction with fatty acids accumulation in NASH pathogenesis remains little investigated.

We used the nutritional model of high fatty acids (HFA), high cholesterol (HCh) or high fat and high cholesterol (HFA+FCh) diets and explored by a lipidomic approach, the blood and liver distribution of fatty acids and oxysterols in response to dietary manipulation. We observed that HFA or HCh diets induced fatty liver without inflammation, which was otherwise observed only after supplementation of HFA+HCh. Very interestingly, the combination model was associated with a specific oxysterol fingerprint.

The present work provides a complete analysis of the change in lipids and oxysterols profile induced by different lipid dietary model and their association with histological alteration of the liver. This study allows the generation of interesting hypotheses on the role of interaction of lipid and cholesterol metabolites in the liver injury during NAFLD development and progression. Moreover, the changes in the concentration and quality of oxysterols induced by a combination diet suggest a novel potential pathogenic mechanism in the progression from simple steatosis to steatohepatitis.

## Highlights

• Dietary fatty acids or cholesterol induce specific liver histological alterations.
• A lipidomic approach allows the identification of steatotic oxysterol fingerprint.
• FA and Ch interaction is a potential pathogenic mechanism for steatohepatitis.

## Graphical abstract

**Figure:**
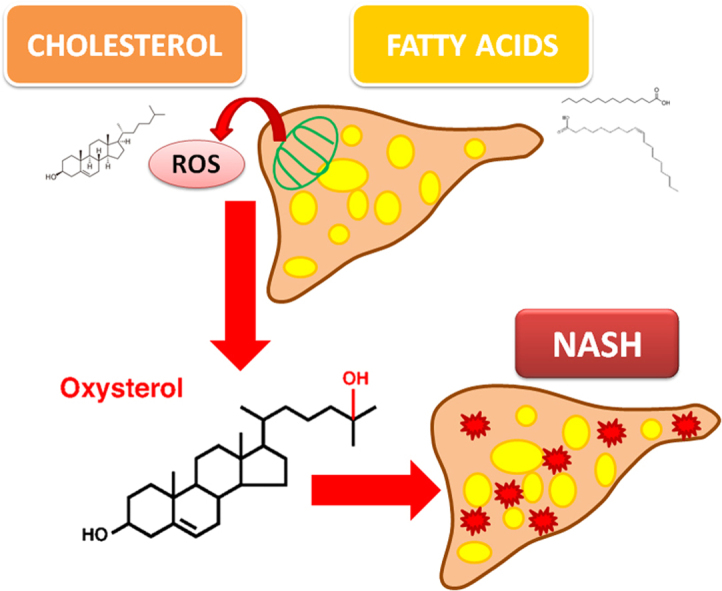

## 1 Introduction

Nonalcoholic fatty liver disease (NAFLD), the most common liver pathology in the Western world [1], covers diverse clinical conditions ranging from benign steatosis (NAFL), steatohepatitis (NASH) – the most progressive form of the disease [2] – through liver cirrhosis. The transformation from non-inflamed to inflamed fatty liver is transient and the underlying mechanisms are not yet elucidated. Potential candidates include alteration in lipid metabolism, mitochondrial dysfunction, inflammation, and oxidative stress [3], [4], [5], [6].

Accumulation of lipids in hepatocytes, the hallmark of NAFLD, is not necessarily dangerous because liver injury is caused by the quality rather than quantity of accumulated lipids. In NAFL, large amounts of triglycerides are accumulated in hepatocytes without harm (8–10). Lipotoxicity defines cellular injury caused by specific and dangerous lipid species [7], i.e. caused by certain fatty acids (FA) and cholesterol-related species.

By using a nutritional model of NAFLD in rat, Matsuzawa et al. [8] showed that the association of a high cholesterol and high FA diet, but not one of them given alone, favored the development of steatohepatitis. These findings indicate that the two lipid species work together in combination in the pathogenesis of steatohepatitis.

Specific FA are important mediators of hepatic lipotoxicity involving multiple mechanisms that activate lysosomal- and mitochondrial-dependent apoptotic pathways, as well as the endoplasmic reticulum stress [9], [10]. On the other hand, certain FA are harmless to the liver. In particular, supplementation of the monounsaturated FA oleic acid leads to safe triglycerides (TG) accumulation in liver cells, while palmitic acid, a saturated fatty acid (SFA), in the same conditions causes apoptosis (8). Similar results can be obtained by manipulating combination of oleic acid and palmitic acid in specific ratios [11].

Excess cholesterol, a leading risk factor in cardiovascular diseases (14), reportedly induce hepatocellular sensitivity to inflammatory mediators [12]. Cholesterol autoxidize to diverse molecules, namely oxysterols, under inflammatory and up-regulated oxidative stress conditions that occurs in the context of liver injury. Having shown potent bioactive properties, oxysterols have been suggested to play a role in NAFLD pathogenesis [13], [14]. Also, oxysterols can be generated enzymatically by cytochrome (CYP) family enzymes, including those channeling oxysterols into the bile acid pathway, whose expression can be altered during liver insult [14], [15].

However, the role of oxysterols and their potential interaction with the FA system in NASH remain little investigated.

In the present work we studied liver damage in rats fed high lipid diets, focusing on supplementation of high FA (HFA), high cholesterol (HCh) or the combination of HFA and HCh enriched diets. We used a lipidomic approach to investigate the distribution of cholesterol, TG, FA, and oxysterols in the liver and in blood in response to the different dietary manipulation.

We observed that HFA or HCh diets induced fatty liver without inflammation and injury, which is otherwise observed after supplementation of HFA plus HCh diet. This diet induced also the highest level of lipid accumulation in the liver, and resulted in a distinct oxysterol fingerprint.

## 2 Materials and methods

### 2.1 Animals and experimental design

All animals received care in compliance with the *Principles of Laboratory Animal Care* formulated by the *National Society for Medical Research and the guide for the Care and Use of Laboratory Animals* prepared by the Institute of Laboratory Animal Resources (NIH Publication No. 86-23, revised 1985), as well as with European Directive on animal experimentation (EU Directive 2010/63/EU for animal experiments).

Male Wistar rats (Harlan Laboratories, San Pietro al Natisone, Italy), 8 weeks old, were housed in colony cages with a 12 h light/12 h dark cycle, and they were fed chow a*d libitum* for 6 weeks. Rats were then allocated into four dietary groups: control (CTRL, n=5), rats were fed chow; HFA (n=5), rats fed high FA (60% cocoa butter); HCh (n=5), rats fed high-cholesterol diet (1.25% cholesterol); and HFA−HCh (n=5), rats fed the combination of high FA and high cholesterol (60% cocoa butter +1.25% cholesterol). The diets were prepared by Mucedola Srl (Settimo Milanese, Italy) according to the levels of components previously reported [8].

Rats were weekly weighted, the amount of chow consumed and the calories introduced were estimated. At the end of the study (6 weeks), animals were anesthetized (100 mg/kg ketamine and 2.5 mg/kg acepromazine i.p.) and then sacrificed, serum and liver harvested. Sections of formalin-fixed, paraffin-embedded samples were stained with haematoxylin/eosin and blinded analyzed by light microscopy for NAFLD activity score [16]. Serum was assayed for alanine aminotransferase (ALT) and aspartate aminotransferase (AST) activity, glucose, total cholesterol, HDL cholesterol, and triglycerides, using standard kits (Sigma Aldric, Milan, Italy).

### 2.2 Fatty acids measurement

Before analysis, hepatic FA were processed for direct transesterification with acetyl chloride according to previously published methods [17], [18], which allowed for the derivatization of both free and esterified FA as methyl esters. Analyses were performed on an Agilent 7820 A Plus Gas Chromatograph (Agilent Technologies) equipped with a G4513A automatic liquid sampler and a flame-ionization detector. Separation was carried out on a 100-m capillary column (Supelco, SP-2560 100 m×0.25 mm inner diameter, 0.20 µm thickness; Sigma Aldrich, Milan, Italy). Identification, precision, and accuracy were evaluated by using mixtures of authentic methylated free FA (FFA) standards and a control pool as previously described [19]. Fatty acids are expressed as the percentage of total fatty acids. An overview of the saturated, monounsaturated, and polyunsaturated fatty acids measured is depicted in Table 1.

### 2.3 Oxysterols measurement

**Fig. 1:**
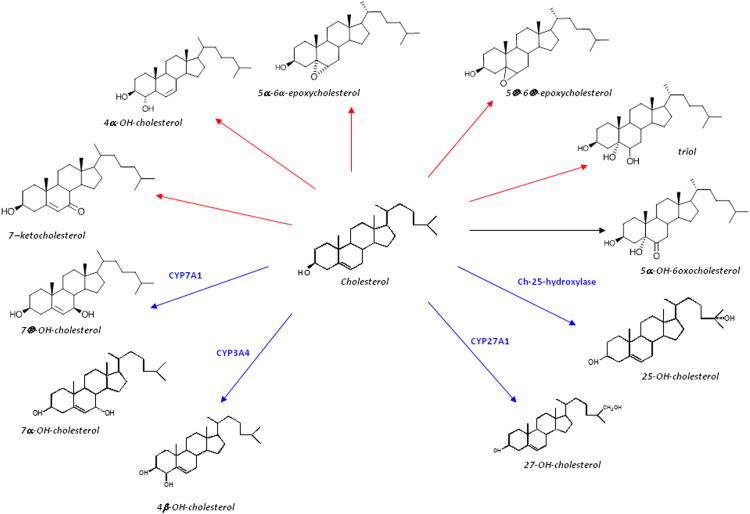
Molecular structure of oxysterols analyzed. Blue lines identify oxysterols of enzymatic origin; red lines identify putative autoxidation oxysterols. For interpretation of the references to color in this figure legend, the reader is referred to the web version of this article.)

7α-hydroxycholesterol (7α-OHC), 7β-hydroxycholesterol (7β-OHC), 27-hydroxycholesterol (27-OHC), 25-hydroxycholesterol (25-OHC), 4β-hydroxycholesterol (4β-OHC), 5β,6β-epoxycholesterol (5β,6β-epoxy), 5α,6α-epoxycholesterol (5α,6α-epoxy), 5α-cholestane-3β,5,6β-triol (triol), 7-ketocholesterol (7-KC) and 6-oxo-cholestan-3β,5α-diol (6-oxo) by mass spectrometry with isotope-dilution methods, as previously reported [20]. Molecular structure is reported in Fig. 1.

### 2.4 Gene expression analysis by real-time RT-PCR

Real-time RT-PCR was performed on RNA extracted from liver tissue, using SYBR Green I assay in Bio-Rad iCycler detection system as previously reported [21]. A PCR master mix containing the specific primers shown in Supplementary Table 1 was used. The threshold cycle (CT) was determined, and the relative gene expression subsequently was calculated as follows: fold change =2^−Δ(ΔCT)^, where ΔCT=CT−CT target housekeeping and Δ(ΔCT)=ΔCT−CT treated control.

### 2.5 Western blot analysis

50 µg proteins from liver homogenates were loaded in a 10% SDS-PAGE and transferred to a nitrocellulose membrane, blocked for 1.5 h using 5% non-fat dry milk in TBS-t and incubated overnight at 4 °C with the following primary antibodies: mouse anti-ABC1 (sc-58219), mouse anti-ABCG5 (sc-517207), rabbit anti-ABCG8 (sc-30111), mouse anti-CYP7A1 (sc-293193), mouse anti-CYP27A1 (sc-393222), rabbit anti-HMGCoA-Reductase (sc-33827), mouse anti-SREBP-2 (sc-271616), mouse anti-FAS (sc-55580), all purchased from Santa Cruz Biotechnology, Heidelberg, Germany). Then, the membrane was incubated for 1.5 h with a rabbit HRP-conjugated anti-mouse or a goat HRP-conjugated anti-rabbit secondary antibody (Bio-Rad Laboratories Inc, Segrate (MI), Italy). Bands were detected by the Clarity™ Western ECL Blotting Substrate using a ChemiDoc MP system (Bio-Rad Laboratories Inc, Segrate (MI), Italy) andquantified by the Image Lab™ Software.

### 2.6 Statistical analysis

The data were normally distributed and were expressed as mean±standard deviation of the mean (SDM). Differences between the groups were determined by one-way analysis of variance (ANOVA) with Tukey-Kramer as *post hoc* test. Statistical significance was accepted when *P*<0.05. The GraphPad Prism 6.0 Software was used to perform the analysis.

## 3 Results

### 3.1 Cholesterol and TG accumulate in serum and liver in association with the development of steatohepatitis

**Fig. 2:**
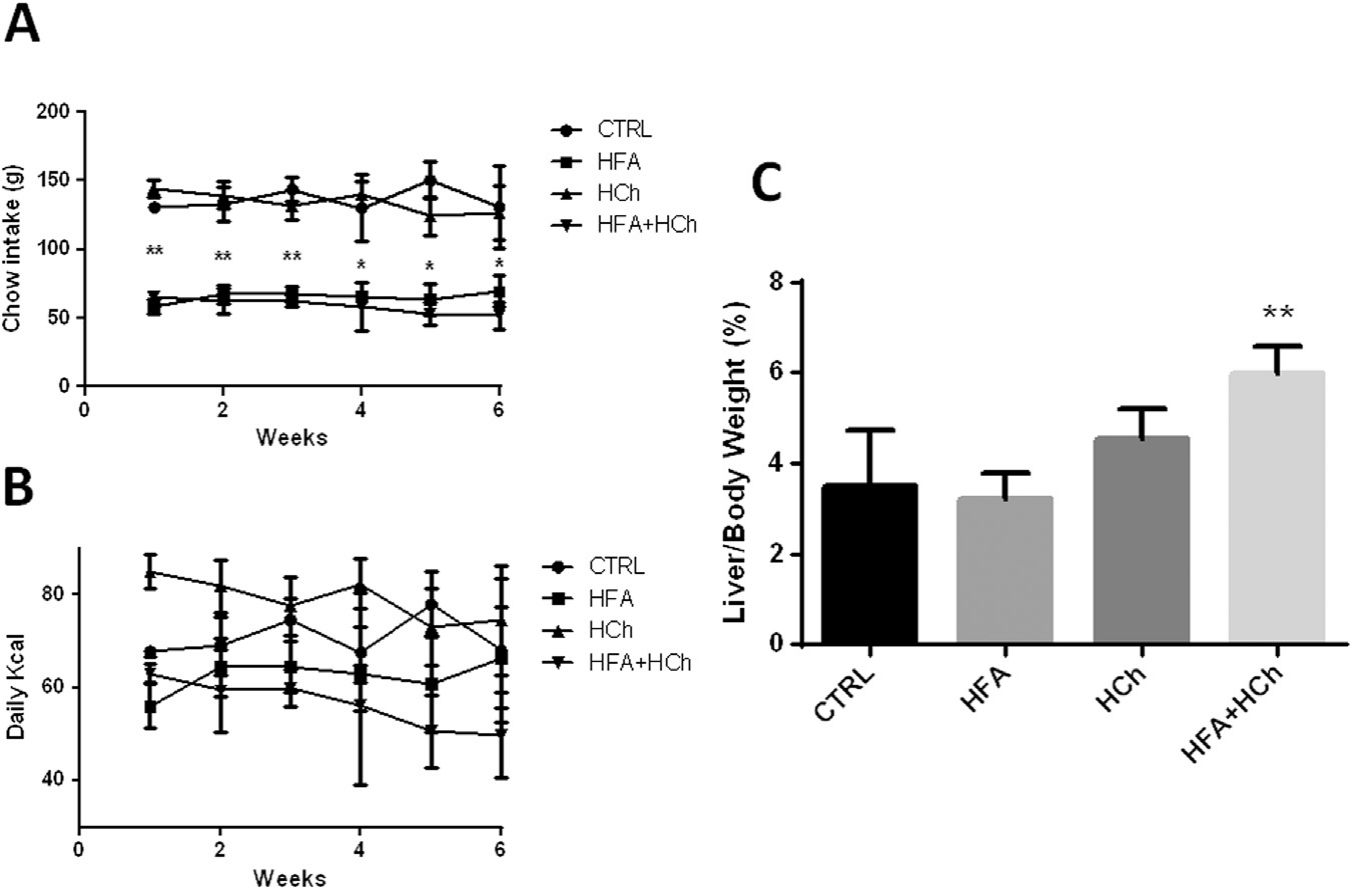
Chow consumed per week (panel A), calories intake (panel B) **r** weight ratio (panel C) in rats fed standard chow (CTRL), high-fat (HFA), high-cholesterol (HCh) and high-fat+high-cholesterol (HFA+HCh) diet for 6 weeks. Data are expressed as mean±SDM. Statistical differences were assessed by one-way ANOVA and Tukey-Kramer as post-hoc test. *=p<0.05 vs CTRL and HFA groups;**=p<0.01 vs CTRL and HFA groups.

Rats from HFA and HFA+HCh groups consumed lower amount of food, compared to CTRL and HCh groups (Fig. 2A); however the calories intake was similar (Fig. 2B). The liver to body weight ratio was significantly increased only in HFA+HCh diet (Fig. 2C).

**Fig. 3:**
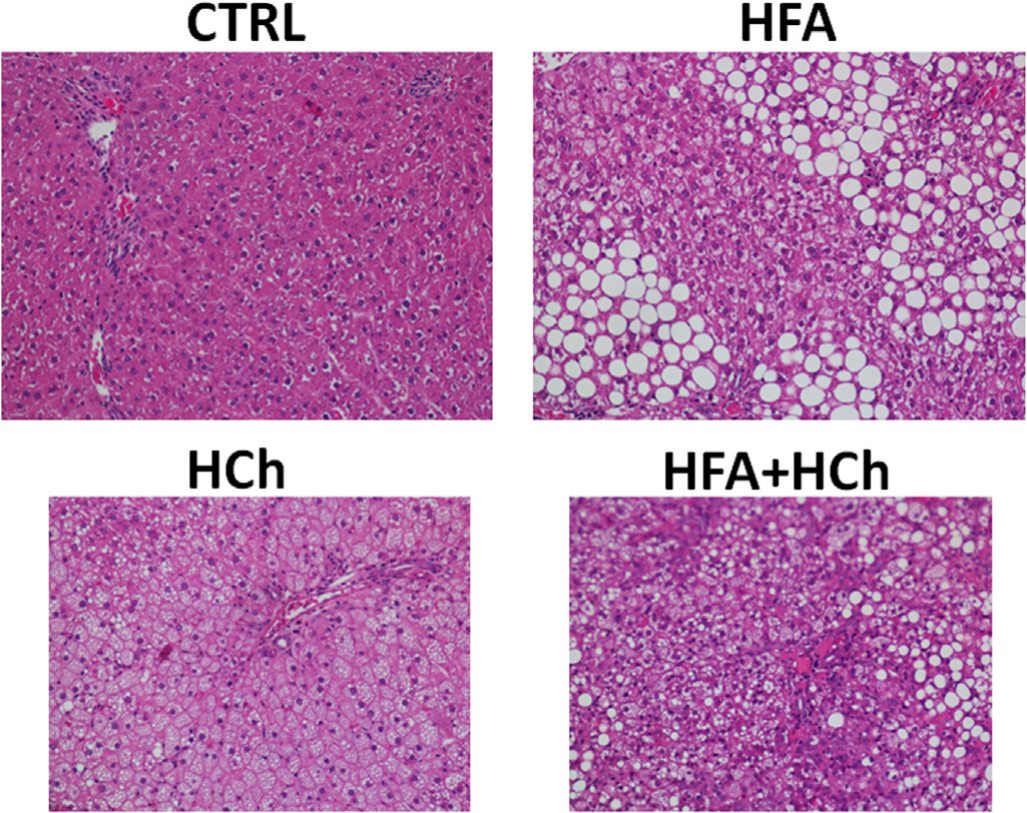
Histological analysis of representative liver samples from rats fed a standard (CTRL), high-fat (HFA), high-cholesterol (HCh) or high-fat+high-cholesterol (HFA+HCh) diet for 6 weeks, stained with Haematoxilin & Eosin (magnification 200x).

Compared to CTRL group, serum liver enzyme levels, including AST, ALT and ALP, resulted significantly higher in HFA+HCh group but not in HFA group, whereas HCh showed significantly altered levels of AST only (Table 2). Liver histology showed massive steatosis but minimal lobular inflammation in HFA group; the liver of rats fed with HCh showed cellular ballooning and steatosis, and mild inflammation; while the liver of HF+HCh group exhibited macrovesicular steatosis, severe hepatocyte ballooning and diffuse lobular infiltration (Fig. 3, Table 3).

Serum cholesterol and LDL-cholesterol were higher in HCh and HFA+HCh groups, and TG was higher in HFA group compared to CTRL. The accumulation of TG in serum observed in HFA group was not observed in HFA+HCh group (Table 4). Concerning cholesterol and TG levels in the liver, both lipids accumulated in the liver of HFA and HFA+HCh groups, and cholesterol but not TG accumulated in the liver of HCh group (Table 4).

### 3.2 Liver FA profiling

**Fig. 4:**
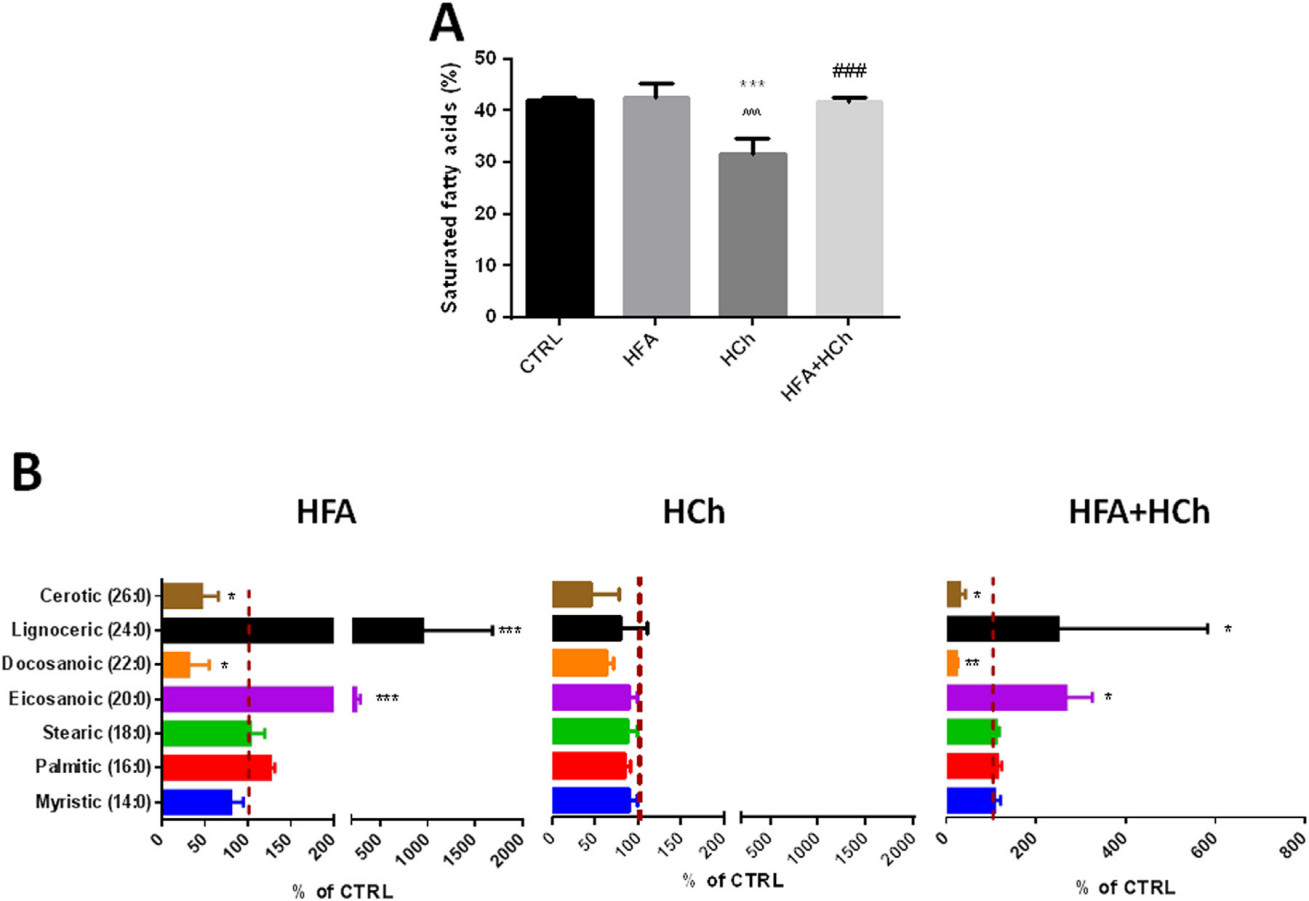
Levels of saturated fatty acids (SFAs, panel A) and complete SFAs profile (panel B) in the liver of rats fed high-fat (HFA), high-cholesterol (HCh) and high-fat+high−cholesterol (HFA+HCh) diet for 6 weeks. Statistical differences were assessed by one-way ANOVA and Tukey-Kramer as post-hoc test (panel A) or by student's *t*-test versus the control group (panel B). *,**,***=p<0.05, 0.01, 0.001 vs CTRL; ^, ^^, ^^^=p<0.05, 0.01, 0.001 vs HFA; #, ##, ###= p<0.05, 0.01, 0.001 vs HCh.

The total level of SFA accumulated in the liver was comparable in HFA and HF+HC group but was significantly lower in HCh, compared to controls. Looking at the distribution of the different SFA accumulated in the liver, we observed a dramatic increase in eicosanoic (20:0) and lignoceric (24:0); and a reduction in docosanoic (22:0) and cerotic acid (26:0) levels in HFA and HFA+HC dietary groups, compared to CTRL (Fig. 4).

**Fig. 5:**
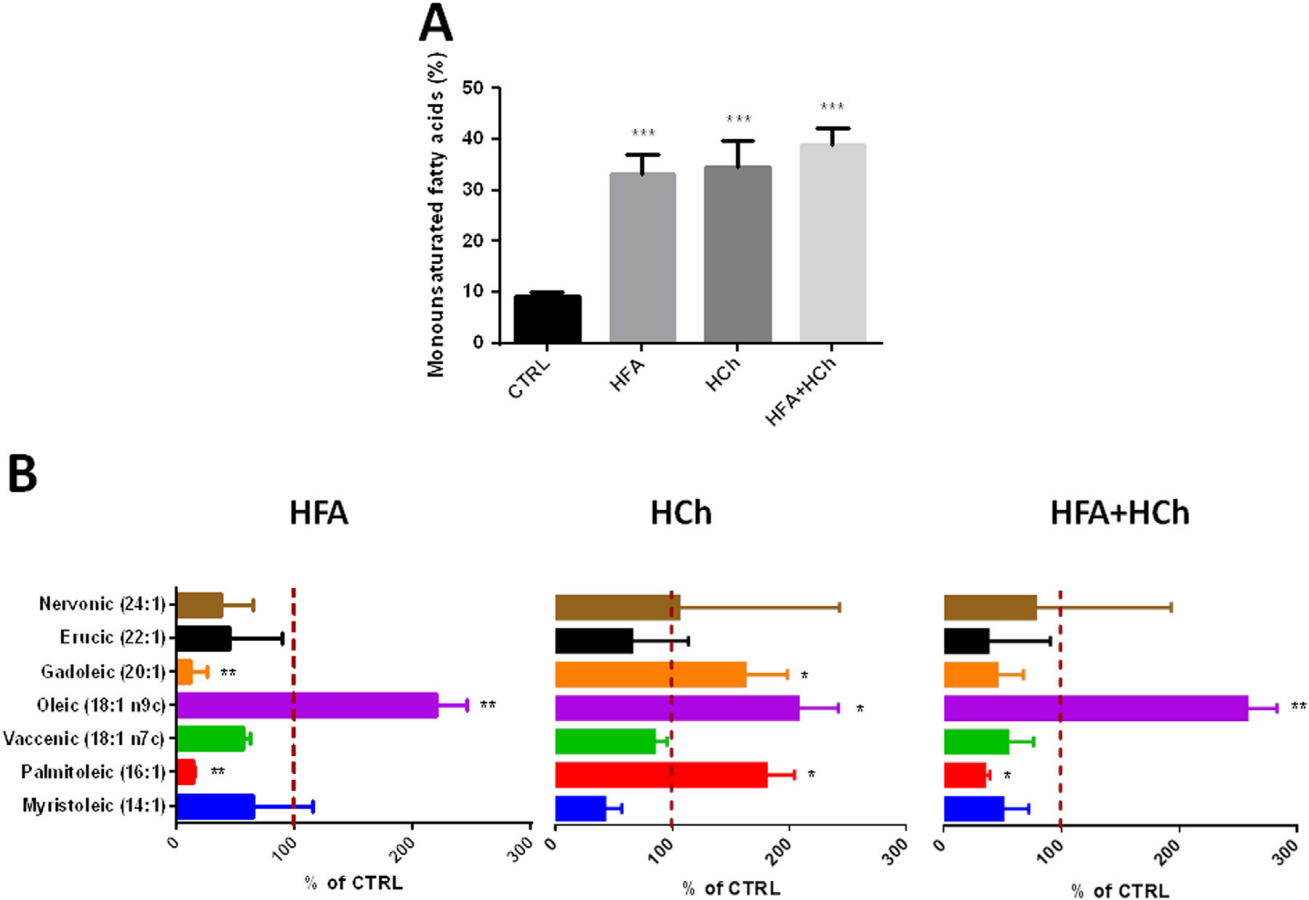
Levels of monounsaturated fatty acids (MUFAs, panel A) and complete MUFAs profile (panel B) in the liver of rats fed high-fat (HFA), high-cholesterol (HCh) and high-fat+high−cholesterol (HFA+HCh) diet for 6 weeks. Statistical differences were assessed by one-way ANOVA and Tukey-Kramer as post-hoc test (panel A) or by student's *t*-test versus the control group (panel B). *,**,***=p<0.05, 0.01, 0.001 vs CTRL; ^, ^^, ^^^=p<0.05, 0.01, 0.001 vs HFA; #, ##, ###=p<0.05, 0.01, 0.001 vs HCh.

The total amount of monounsaturated FA (MUFA) accumulated in the liver was larger in all three dietary regimens, compared to chow, with a consistent accumulation of oleic acid (18:1 n-9 *cis*). The liver of HFA and HFA+HCh groups showed reduced levels of palmitoleic (16:1 n-7 *cis*) and gadoleic acid (20:1 n-11 *cis*). An altered pattern of MUFA was also observed in HCh fed rats, compared to chow, with increased levels of palmitoleic and gadoleic acid (Fig. 5).

The total amount of polyunsaturated FA (PUFA) was reduced in all three dietary regimens, compared to chow. In HFA, higher levels of gamma-linolenic (18:2n-6 *cis*), adrenic (22:4n-6 *cis*) and docosapentanoic acid (22:5n-3 *cis*), contrasted with lower levels of eicosadienoic (20:2n-6 *cis*), docosadienoic (22:2n-6 *cis*) and arachidonic acid (20:4n-6 *cis*). In the liver of HFA+HCh the distribution of ω-6 FA was similar to that of HFA diet, but with lower levels of linoleic acid (18:2n-6 *cis*). In HCh diet ω-6 profiling revealed a reduction in gamma-linolenic (18:2n-6 *cis*), eicosadienoic (20:2n-6 *cis*) and docosadienoic acid (22:2n-6 *cis*).

Concerning PUFA of ω-3 series, a lower content of linolenic acid (18:3n-3 *cis*), eicosatrienoic acid (20:3n-3 *cis*), eicosapentaenoic acid (20:5n-3 *cis*) and docosapentaenoic acid (22:5n-3 *cis*) was observed in HFA group; eicosapentaenoic acid (20:5n-3 *cis*) and docosahexaenoic acid (22:6n-3 *cis*) were reduced in HFA+HCh group, and a significant increase in eicosapentaenoic acid (20:5n-3 *cis*) was observed in HCh group.

**Fig. 6:**
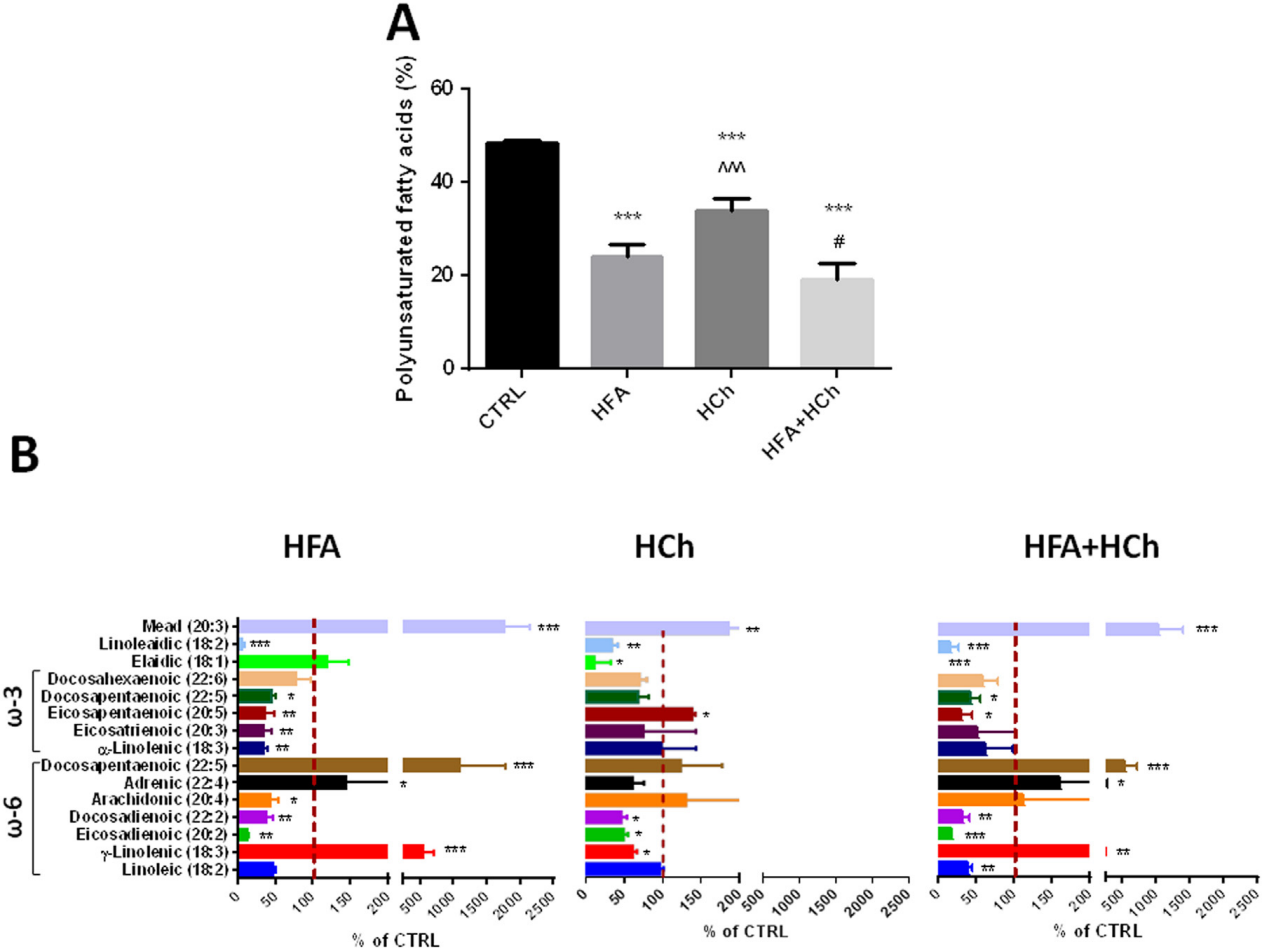
Levels of polyunsaturated fatty acids (PUFAs, panel A) and complete PUFAs profile (panel B) in the liver of rats fed high-fat (HFA), high-cholesterol (HCh) and high-fat+high−cholesterol (HFA+HCh) diet for 6 weeks. Statistical differences were assessed by one-way ANOVA and Tukey-Kramer as post-hoc test (panel A) or by student's *t*-test versus the control group (panel B). *,**,***=p<0.05, 0.01, 0.001 vs CTRL; ^, ^^, ^^^=p<0.05, 0.01, 0.001 vs HFA; #, ##, ###=p<0.05, 0.01, 0.001 vs HCh.

All groups showed accumulation of mead acid (20:3n-9 *cis*). Trans-FA elaidic acid (18:1n-9 *trans*) was reduced in HCh and HF+HCh groups, and linoleaidic acid (18:2n-9 *trans*) was reduced in all three dietary groups, compared to chow (Fig. 6).

### 3.3 Liver oxysterol profiling

**Fig. 7:**
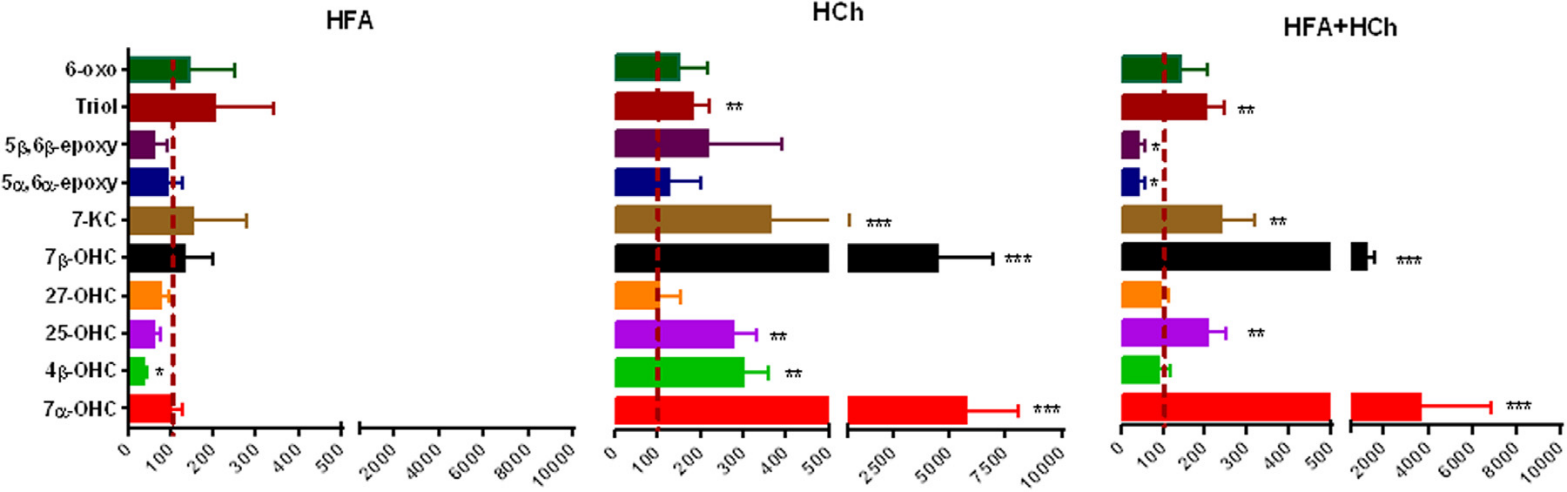
Oxysterol profile in the liver of rats fed high-fat (HFA), high-cholesterol (HCh) and high-fat+high−cholesterol (HFA+HCh) diet for 6 weeks. Data are expressed as mean ± SDM of the percentage relative to the levels of rats fed the standard chow (CTRL). Statistical differences were assessed by the student's *t*-test. 7α-OHC, 7α-hydroxycholesterol; 7β-OHC, 7β-hydroxycholesterol; 27-OHC, 27-hydroxycholesterol; 25-OHC, 25-hydroxycholesterol; 4β-OHC, 4β-hydroxycholesterol; 5α,6α-epoxy, 5α,6α-epoxycholesterol; 5β,6β-epoxy, 5β,6β-epoxycholesterol; triol, 5α-cholestane-3β,5,6β-triol; 7-KC, 7-ketocholesterol; 6-oxo, 6-oxo-cholestan-3β,5α-diol.

No significant changes in liver oxysterol content was observed in response to HFA diet, with exception of reduced levels of 4β-hydroxycholesterol. HCh diet induced accumulation of 7α-, 7β-, 4β-, 25-hydroxycholesterol, 7-ketocholesterol, and triol. This pattern was not conserved in the combined high lipid diets (HFA+HCh), in that 4β-hydroxycholesterol unchanged and 5α,6α- and 5β,6β-epoxides were reduced compared to chow (Fig. 7).

### 3.4 Expression of genes/proteins involved in cholesterol metabolism and fatty acid synthesis

Cholesterol oxidation involves several mono-oxygenation reactions, which are catalyzed by cytochromes P450 (CYPs). During normal hepatic cholesterol degradation, cholesterol can be enzimatically converted to 7α-hydroxycholestrol (7α-OHC) by 7α-hydroxylase (CYP7A1). Other CYPs such as 27-hydroxylase (CYP27A1), 25-hydroxylase (CYP25A1) and 4β-hydroxylase (CYP3A1) catalyze the formation of the oxysterol species 27-hydroxycholesterol (27-OHC), 25-hydroxycholesterol (25-OHC) and 4β-hydroxycholestrol (4β-OHC), respectively. Both 7α-OHC and 27-OHC are the first intermediates in the biosynthesis of bile acids [13]; however, while 7α-OHC initiates the canonical pathway, 27-OHC is the first product of the alternative pathway for bile acids synthesis [13].

Overexpression of CYP7A1 accounted for the increased 7α-OHC concentration observed in HCh and HFA+HCh diets whereas CYP27A1 was unchanged.

When cholesterol accumulates in the hepatocytes, proteins deputed to cholesterol secretion such as ABCA1*,* ABCG5 and ABCG8 are activated. In fact, we observed up-regulation of such proteins in cholesterol containing diets.

**Fig. 8:**
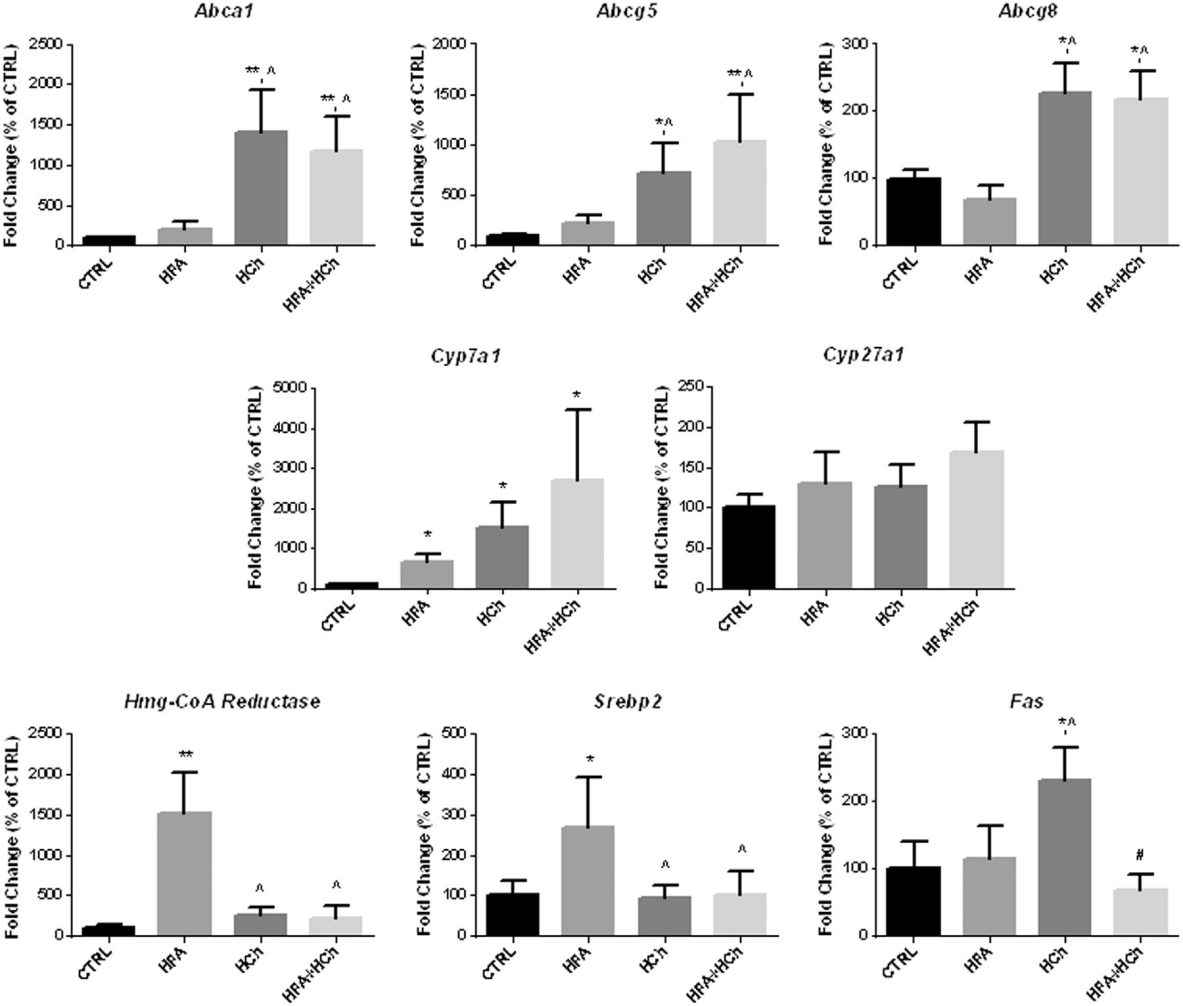
Expression levels of genes involved in cholesterol and fatty acids metabolism in the liver of rats fed standard chow (CTRL), high-fat (HFA), high-cholesterol (HCh) and high-fat+high−cholesterol (HFA+HCh) diet for 6 weeks. Data are expressed as mean±SDM of the percentage of the fold change expression related to CTRL. Statistical differences were assessed by one-way ANOVA and Tukey-Kramer as post-hoc test. *,**=p<0.05, 0.01 vs CTRL; ^=p<0.05 vs HFA.

**Fig. 9:**
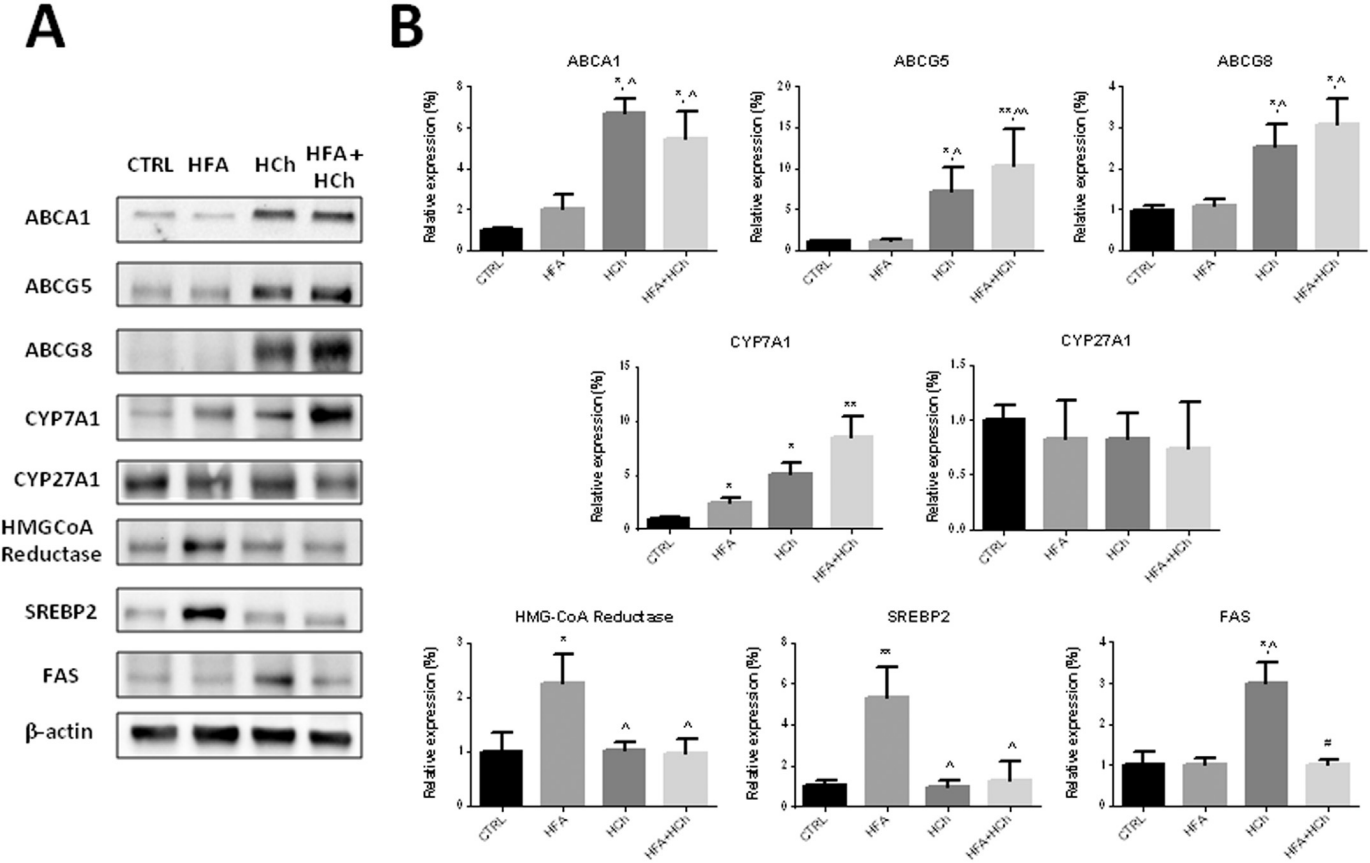
Representative blots (panel A) and relative densitometric analysis (panel B) of proteins involved in cholesterol and fatty acids metabolism in the liver of rats fed standard chow (CTRL), high-fat (HFA), high-cholesterol (HCh) and high-fat+high−cholesterol (HFA+HCh) diet for 6 weeks. Data are expressed as mean±SDM. Statistical differences were assessed by one-way ANOVA and Tukey-Kramer as post-hoc test. *,**=p<0.05, 0.01 vs CTRL; ^=p<0.05 vs HFA.

In the HFA diet we observed activation of the cholesterol synthesis (increased HMG-CoA reductase and SREBP2 expression) that was not observed in HCh diet where synthesis of fatty acids was up-regulated. Interestingly, both cholesterol and fatty acids synthesis, that are part of the strategy to limit accumulation of FFAs and cholesterol, did not activate in the combination diet (Fig. 8, Fig. 9).

## 4 Discussion

NAFLD is characterized by altered lipid homeostasis, but there is little data on the potential interaction of various lipid classes. i.e. fatty acids and cholesterol metabolism, during NAFLD development and progression. Puri et al. reported that FFA content in the liver remains unaltered during NAFLD development despite an increase in the total lipid content [22]. They also observed a progressive increase in free cholesterol and a decrease in phospholipid content during disease evolution, from simple steatosis through NASH, suggesting a complex interaction among different lipids in the pathogenesis of liver metabolic disease.

From the first observation of Matsuzawa reporting that the combination of FA and cholesterol in a high lipid diet is associated with the induction of steatohepatitis [8], nutritional models have been proposed to explore the complex change occurring not only in the amount but rather in the quality of different lipotoxic products during steatohepatitis progression. A novel and intriguing role has been suggested for the oxidative products of cholesterol, namely oxysterols, which are produced by enzymatic mechanisms or by autoxidation [23], [24]. However, so far no information have been provided on the “cholesterol side” of the lipid changes induced by different hyper-lipidic diets.

Based on standardized animal models, we provide evidence that specific histological modifications in the liver, observed with different lipid diets, associate with altered FFAs and oxysterols profile. Our data confirm previous observation that high fat diet induces only a non-progressive steatosis due to triglycerides accumulation whereas high cholesterol diet causes hepatocyte ballooning due to cholesterol accumulation. Importantly, only the combination of cholesterol and fatty acids in the high lipid diet resulted steatosis, cellular ballooning and inflammation.

The HFA-HCh diet was associated with significant differences in the composition of saturated, monounsaturated and polyunsaturated fatty acids content in the liver. In particular, even though the total level of SFAs was similar to the controls – as occurring in human pathology [22], [25] – long chain SFAs accumulated in high fat-based diets but not in the high cholesterol alone.

The FA and cholesterol dietary content also impacted in the composition of hepatocytes lipids in terms of MUFA and PUFA; even though MUFA increased in the HFA and HFA+HCh diets, we observed only a significant increase in the oleic acid level, the most abundant MUFA, whereas palmitoleic (16:1n-7) and gadoleic (20:1n-11) acids increased only in the cholesterol diet. The analysis of PUFA showed that ω-6 was lower in high fat based diets as compared to cholesterol diet whereas change in the ω-3 amount was less evident. Very interestingly, arachidonic acid (20:4n-6) was reduced in the high fat group while it was highly variable in the combination diet and associated with a reduction of n-6 precursor (linoleic acid). Eicosapentaenoic acid (20:5n-3) and docosahexaenoic acid (22:6n-3) were also reduced in HFA and HFA+HCh diets but not in the HCh diet. The reduction of n-3 precursor and the increase in mead acid (20:3n-9) level, particularly in the combination diet, indicate that this is not due to a deficiency in availability of essential fatty acids (EFAs) but rather to an inefficiency in the conversion of these precursor EFAs to downstream ω-3 and ω-6 fatty acids, in good agreement with the observation in man [22]. Our data also suggest that high fat diet induces a different regulation of the desaturation index (lower palmitoleic/palmitic and higher oleic/stearic), probably related to increased chain elongation [26]. The control of desaturase signaling and the role played in the liver injury merit further investigations.

Even more intriguing are the results of oxysterol profile. Oxysterols are metabolites of cholesterol that are produced in the liver and other peripheral tissues as a means to eliminate cholesterol to bile acid. During normal hepatic cholesterol degradation, cholesterol can be enzimatically converted to 7α-hydroxycholesterol (7α-OHC) by 7α-hydroxylase (CYP7A1). Our data show that in the high cholesterol based diets 7α-OHC dramatically increased as a consequence of overexpression of CYP7A1 that is the main regulator of the canonical pathway of bile acids synthesis (16). On the contrary, other CYPs such as 27-hydroxylase (CYP27A1), 25-hydroxylase (CYP25A1) and 4β-hydroxylase (CYP3A1) catalyze the formation of 27-hydroxycholesterol (27-OHC) (the most abundant circulating oxysterol), 25-hydroxycholesterol (25-OHC) and 4β-hydroxycholesterol (4β-OHC), respectively. Both 7α-OHC and 27-OHC are the first intermediates in the biosynthesis of bile acids [13]; however, while 7α-OHC initiates the canonical pathway, 27-OHC is the first product of the alternative pathway for bile acids synthesis [13]. In human NASH, it has been reported a shift from the canonical to the alternative bile acids synthesis pathway as a mechanism to limit hepatotoxicity of cholesterol [27]. It is worth to note that the alternative pathway becomes more predominant and compensates for limitations of the classical pathway during liver disease [28]. Here, we observed an increase of 7α-OHC, together with a higher expression of *Cyp7a1*, in the liver of high cholesterol diets, while no modifications occurred for 27-OHC concentration and *Cyp27a1* expression. Accordingly, the excess dietary cholesterol induced activation of the canonical but not the alternative pathway of bile acids synthesis.

25-OHC reportedly acts as a signal to suppress cholesterol synthesis through the degradation of SREBPs and HMG-CoA reductase [29]. Our data show that, during high fat diet, fatty acids synthesis is inhibited and cholesterol synthesis is conserved as a mechanism of fatty acids elimination. In high cholesterol diet, *Fas* is overexpressed and cholesterol synthesis inhibited to shift cholesterol accumulation toward fatty acids; in the combination diet, both *Fas* and *Hmg-CoA reductase* are inhibited: as a consequence, both fatty acid and cholesterol excess cannot be eliminated.

Cholesterol can be alternatively subjected to nonenzymatic autoxidation, which leads to the formation of a number of cholesterol oxidation products, including 7-ketocholesterol (7-KC), 7β-hydroxycholesterol (7β-OHC), 5α,6α-epoxycholesterol (5α,6α-epoxy), 5β,6β-epoxycholesterol (5β,6β-epoxy), 5α-cholestane-3β,5,6β-triol (triol), and 6-oxo-cholestan-3β,5α-diol (6-oxo). The modification in the metabolic pathway associated with different oxysterol profile: in the HF diet, enzymatic oxysterols were unchanged and among the non-enzymatic, epoxides levels were conserved with an increase in Triol and 6-oxo; in the high-cholesterol diet the non-enzymatic 7-KC and 7β-OHC increase dramatically together with 25-OHC, 4β-OHC and 7α-OHC as possible signaling strategy to eliminate cholesterol excess. In the combination diet, 4β-OHC was unchanged and epoxides were low as compared to HCh and HFA diet: this probably accounted, almost in part, for the inefficient cholesterol and fatty acid elimination. Since the accumulation of 4β-OHC may activate inflammation, this may explain mild inflammation in HC livers as compared to HF [30].

The dietary cholesterol excess leads to increased hepatic levels of 7β-OHC and 7-KC, as reported in both the HCh and the HFA+HCh groups. Since such compounds are considered cytotoxic and pro-apoptotic [13], we hypothesize that their accumulation in the liver, together with FFAs, might take part in the pathogenic mechanism of the transition from simple steatosis to steatohepatitis.

The role of epoxides is under active investigation since the formation of 5β,6β-epoxy has been observed previously as a result of cholesterol peroxidation and autoxidation [31]. This oxysterol was shown to initiate cellular apoptosis in several systems and to play a role in lipid loading of macrophages [32], [33], [34]. Moreover, their role has been previously suggested in the damage of lung epithelial cells exposed to ozone [35]. Both 5β,6β-epoxy and 6-oxo are potent inhibitors of cholesterol synthesis from acetate, a pathway that involves numerous steps and several intermediates. Such oxysterols may interfere with the prenylation of proteins, and it has been suggested that blocking protein prenylation can cause changes in inflammatory signaling [36]. The reason why in the combination diet the level of β-epoxides decreases is not known, but their capacity to inhibit cholesterol synthesis suggests a possible role in the hepatic injury induced by cholesterol accumulation.

The present study was performed on male Wistar rats and conclusions cannot be generalized to female; this may be a possible limitation, since a genre difference in dietary lipid manipulation may exist, in particular this is valid for metabolic syndrome in humans [37].

In conclusion, the present work provides a complete analysis of the change in lipids and oxysterols profile induced by different lipid dietary model and their association with histological alteration of the liver. Even though the biological implications of the reported changes in the lipid composition are complex and difficult to predict simply on the basis of lipidomic data, the present study allows the generation of interesting hypotheses on the role of interaction of lipid and cholesterol metabolites in the liver injury during NAFLD development and progression. Moreover, the changes in the concentration and quality of oxysterols induced by a combination diet suggest a novel potential pathogenic mechanism in the progression from simple steatosis to steatohepatitis.

## Competing interests

The authors declare no competing interests.

## Figures and Tables

**Table 1:** An overview of the fatty acid profile analyzed in the study. Both the trivial and the IUPAC names are reported, together with the short-hand nomenclature, which designates the fatty acid carbon chain length and the number of double bonds. Of the polyunsaturated fatty acids, linoleic acid and α-linolenic acid are also known as essential fatty acids because they are necessary and cannot be synthesized in the body.

**Abbreviation**	**Trivial name**	**International Union of Pure and Applied Chemistry name**
C14:0	Myristic acid	Tetradecanoic acid
C16:0	Palmitic acid	Hexadecanoic acid
C18:0	Stearic acid	Octadecanoic acid
C20:0	Arachidic acid	Icosanoic acid
C22:0	Behenic acid	Docosanoic acid
C24:0	Lignoceric acid	Tetracosanoic acid
C26:0	Cerotic acid	Hexacosanoic acid
C14:1n-5	Myristoleic acid	(9*Z*)-Tetradec-9-enoic acid
C16:1n-7	Palmitoleic acid	(9*Z*)-Hexadec-9-enoic acid
C18:1n-7	Vaccenic acid	(11Z)-Octadec-11-enoic-acid
C18:1n-9	Oleic acid	(9*Z*)-Octadec-9-enoic acid
C20:1n-9	Gondoic acid	(11Z)-Eicos-11-enoic acid
C22:1n-9	Erucic acid	(13Z)-Docos-13-enoic acid
C24:1n-9	Nervonic acid	(15Z)-Tetracos-15-enoic acid
C18:2n-6	Linoleic acid	(9Z,12Z)-Octadeca-9,12-dienoic acid
C18:2n-6	γ-linolenic acid	(6Z,9Z,12Z)-Octadeca-6,9,12-trienoic acid
C20:2n-6	Eicosadienoic acid	(11Z,14Z)-Icosa-11,14-dienoic acid
C20:3n-6	Dihomo-γ-linolenic acid	(8Z,11Z,14Z)-Icosa-8,11,14-trienoic acid
C20:4n-6	Arachidonic acid	(5Z,8Z,11Z,14Z)-Icosa-5,8,11,14-tetraenoic acid
C22:2n-6	Docosadienoic acid	(13Z,16Z)-Docosa-13,16-dienoic acid
C22:4n-6	Adrenic acid	(7Z,10Z,13Z,16Z)-Docosa-7,10,13,16-tetraenoic acid
C22:5n-6	Osbond acid	(4Z,7Z,10Z,13Z,16Z)-Docosa-4,7,10,13,16-pentaenoic acid
C18:3n-3	α-linolenic acid	(9Z,12Z,15Z)-octadeca-9,12,15-trienoic acid
C20:3n-3	Eicosatrienoic acid	(11Z,14Z,17Z)-eicosa-11,14,17-trienoic acid
C20:5n-3	Eicosapentaenoic acid	(5Z,8Z,11Z,14Z,17Z)-eicosa-5,8,11,14,17-pentaenoic acid
C22:5n-3	Docosapentaenoic acid	(7Z,10Z,13Z,16Z,19Z)-docosa-7,10,13,16,19-pentaenoic acid
C22:6n-3	Docosahexaenoic acid	(4Z,7Z,10Z,13Z,16Z,19Z)-Docosa-4,7,10,13,16,19)-hexaenoic acid
		

C20:3n-9	Mead acid	(5Z,8Z,11Z)-Eicosa-5,8,11-trienoic acid
C18:1n-9t	Elaidic acid	(9E)-Octa-9-decenoic acid
C18:2n-6t	Linoleadic acid	(9E,12E)-octadeca-9,12-dienoic acid

**Table 2:** Serum aminotransferases from rats fed a standard (CTRL), high-fat (HFA), high-cholesterol (HCh) or high-fat+high−cholesterol (HFA+HCh) diet. AST, aspartate aminotransferase; ALT, alanine aminotransferase; ALP, alkaline phosphatase. Data are expressed as mean±SDM. Statistical differences were assessed by one-way ANOVA and Tukey-Kramer as post-hoc test. *,**,***=p<0.05, 0.01, 0.001 vs CTRL; ^, ^^, ^^^=p <0.05, 0.01, 0.001 vs HFA; #, ##, ###=p<0.05, 0.01, 0.001 vs HCh.

	**CTRL**	**HFA**	**HCh**	**HFA+HCh**
**Serum AST (U/L)**	48.0.± 2.9	54.6±1.7	218.5±90.9*,^	428.0±106.1**,^^,^**#**^
**Serum ALT (U/L)**	47.0±6.2	44.6±9.9	137.8±102.1	389.7±147.1*,^
**Serum ALP (U/L)**	198.0±104.1	212.4±71.4	186.5.±54.7	452.0±104.9*,^,^**##**^

**Table 3:** Kleiner scoring system applied to liver samples from rats fed a standard (CTRL), high-fat (HFA), high-cholesterol (HCh) or high-fat+high−cholesterol (HFA+HCh) diet.

	**CTRL**	**HFA**	**HCh**	**HFA+HCh**
**Steatosis**	0	3	1	2
**Ballooning**	0	0	3	2
**Lobular inflammation**	0	1	1	2
**Activity score**	0	4	5	6
**Indication**	Normal	NAFL	NASH	NASH

**Table 4:** Serum biochemistry and liver lipids content from rats fed a standard (CTRL), high-fat (HFA), high-cholesterol (HCh) or high-fat+high−cholesterol (HFA+HCh) diet. AST, aspartate aminotransferase; ALT, alanine aminotransferase; ALP, alkaline phosphatase. Data are expressed as mean±SDM. Statistical differences were assessed by one-way ANOVA and Tukey-Kramer as post-hoc test. *,**,***=p<0.05, 0.01, 0.001 vs CTRL; ^, ^^, ^^^=p<0.05, 0.01, 0.001 vs HFA; #, ##, ###=p<0.05, 0.01, 0.001 vs HCh.

	**CTRL**	**HFA**	**HCh**	**HFA+HCh**
**Serum Glucose (mg/dL)**	157.7±41.1	193.7±50.1	155.3±50.1	116.3±14.8
**Serum Total Cholesterol (mg/dL)**	64.0±4.6	59.0±12.2	195.0±30.5***,^^^	195.5±32.9***,^^^
**Serum LDL-Cholesterol (mg/dL)**	20.0±3.6	7.7±2.9	146.2±9.8***,^^^	100.0±10.9***,^^^
**Serum Triglycerides (mg/dL)**	55.0±14.1	92.3±14.0*	62.0±14.7	66.0±9.3
**Hepatic Triglycerides (µg/mg prot)**	65.3±14.2	132.3±21.3**,^**#**^	90.2±20.3	152.9±19.9***,^**##**^
**Hepatic Total Cholesterol (µg/mg prot)**	40.9±4.4	162.3±24.5***	190.7±30.2***	332.4±39.1***,^^^,^**###**^
