# Supplementary material for: Effects of dietary fatty acids and cholesterol excess on liver injury: A lipidomic approach
Source: Redox Biol. 2016 Sep 9;9:296–305. doi: 10.1016/j.redox.2016.09.002 (PMC5026694; doi:10.1016/j.redox.2016.09.002)
Supplement: Supplementary file 1 — Supplementary material [file mmc1.docx]

| **Gene** | **Direction** | **Primer sequence (5’ to 3’)** |
| --- | --- | --- |
| **Cyp7A1** | Forward | CTG CGA AGG CAT TTG GAC AC |
|  | Reverse | ACC CAG GCA TTG CTC TTT GA |
| **Cyp27A1** | Forward | ACA CAT CCT GAT TGG AAG GGG |
|  | Reverse | GTC TCA TGC GGC TCA ACA CA |
| **Abcg8** | Forward | GCT CAT TCC TTA CCC ACC CTC |
|  | Reverse | TTT GTC GGA GTC CCC AGT GA |
| **Abcg5** | Forward | TAG GTA GCT CAG GCT CAG GGA AA |
|  | Reverse | CCA GCA TCG CCG TGT ATC T |
| **Abca1** | Forward | GAA TGT CCT GCG ATC ACC CA |
|  | Reverse | AGG GGA ACG ACT GAC AGA CT |
| **Fas** | Forward | AGC CTG AGC TTG TCC CTA GA |
|  | Reverse | CAC TGG TAC ACT TTC CCG CT |
| **Srebp2** | Forward | CCG AAC TGG GCG ATG GAT G |
|  | Reverse | TGT AGC ATC TCG TCG ATG TCC |
| **HmgCoAR** | Forward | CCT CCA TTG AGA TCC GGA GG |
|  | Reverse | AAG TGT CAC CGT TCC CAC AA |

**Supplementary Table 1.** Primer sequences for quantitative real-time PCR.
